# Pool Toes: Case Report and Review of Pool-Associated Pedal Dermatoses

**DOI:** 10.7759/cureus.11756

**Published:** 2020-11-28

**Authors:** Philip R Cohen

**Affiliations:** 1 Dermatology, San Diego Family Dermatology, National City, USA

**Keywords:** aquatic, beach, dermatoses, feet, fresh, pedal, pool, swimming, toes, water

## Abstract

Pool swimmers are susceptible to pedal dermatoses. The causes for these conditions are asteototic dermatitis, contact dermatitis, friction-induced injury, infections, keratoderma, pruritus, thermal injury, ultraviolet radiation exposure, and urticaria. The specific dermatoses include aquagenic keratoderma, aquagenic pruritus, aquagenic urticaria, cold-induced urticaria, contact urticaria, erosion interdigitalis blastomycetica, erythrasma, Mycobacterium abscessus hand and foot disease, onychomycosis, photosensitivity, pitted keratolysis, plantar verruca, pool dermatitis, pool feet, pool toes, pool water dermatitis, pseudomonas hot-foot syndrome, skin cancer, subcorneal hematoma, sunburn, swim fin dermatitis, tinea pedis, toe web infection, and xerosis. A seven-year-old girl developed erythema and superficial blisters on her feet after they were exposed to the surface of her new swimming pool. The friction-induced injury, referred to as pool toes, resulted in skin lesions not only on the plantar surface of her toes but also on the areas of her soles-including her heels-that came in contact with the rough pool surface. In addition to discussing the characteristics of pool toes, the features of the other pool-associated pedal dermatoses are reviewed.

## Introduction

The occurrence of some cutaneous conditions is idiopathic; an associated triggering factor is not readily apparent. However, some skin disorders occur more commonly in particular individuals such as athletes. In addition, the environment in which the sports enthusiast participates may influence the dermatoses that develop [1].

Aquatic sports occur in either freshwater or saltwater. Lakes, ponds, and swimming pools are common sources of freshwater. Participants of aquatic sports in the freshwater environment may develop activity-related skin conditions [1].

A seven-year-old girl developed pool toes following her initial exposure to the bottom surface of a newly constructed swimming pool in her backyard. The friction-induced injury resulted in early blister formation not only on the plantar surface of her toes but also on the areas of her soles--including her heels--that came in contact with the rough swimming pool surface. In addition to discussing the characteristics of pool toes, the features of other pool-associated pedal dermatoses are reviewed [1-20].

## Case presentation

A seven-year-old girl presented with painful red feet. Her feet had become tender and erythematous during the several hours of swimming and playing in a newly constructed freshwater pool; indeed, this was the first time she had been in the pool, and she had never developed similar pedal symptoms or lesions when she swam in other pools. When she finished her aquatic activities, she had difficulty walking on her tender feet.

The pool’s bottom surface had been finished with a non-slip surface. It was created by mixing small pebbles with cement. It was rough.

**Figure 1:**
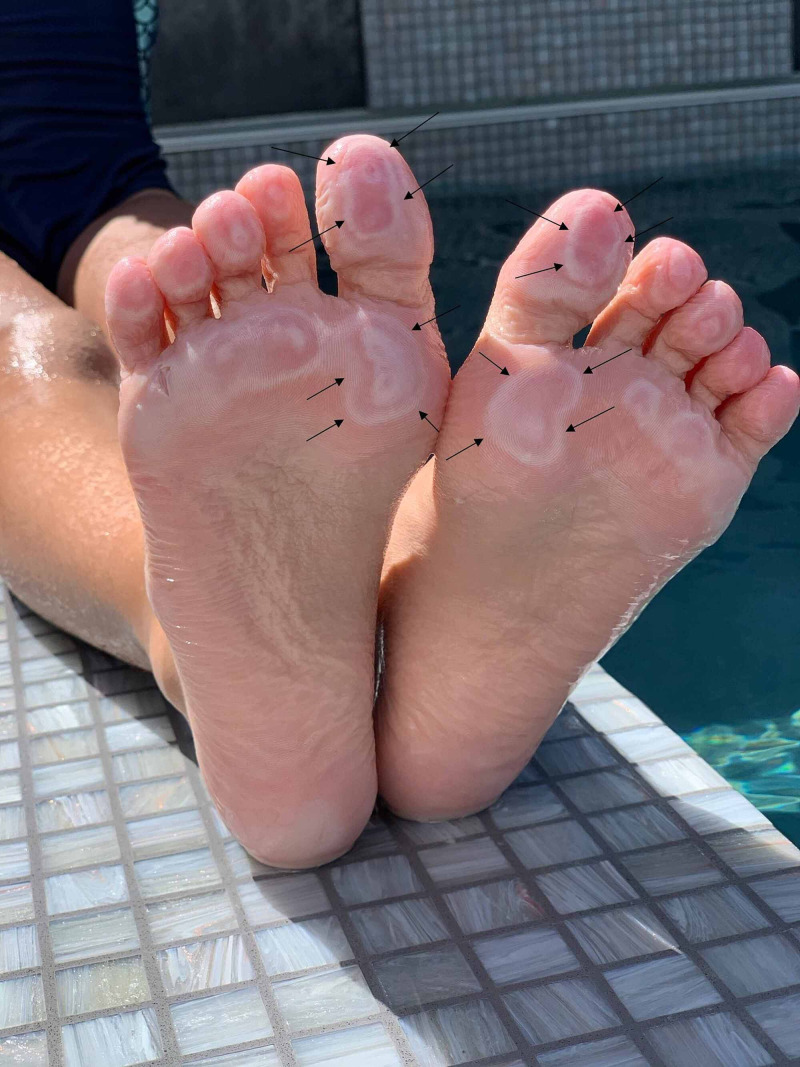
Pool toes on the soles of a seven-year-old girl The plantar surface of the toes and the feet of a seven-year-old girl who experienced a friction-induced injury to the affected areas by repetitively rubbing the soles on the rough bottom of the swimming pool. The contact sites between each foot and the rough pool bottom demonstrate early blister formation presenting as tender central erythema, which is surrounded by annular white areas (black arrows).

Cutaneous examination of her plantar feet showed early blister formation presenting as tender central erythema, which is surrounded by annular white areas. The lesions were located on the skin overlying her metatarsals and distal phalanges and, to a lesser degree, the heel of the ventral feet (Figure 1). These were the sites that had come in contact with the bottom surface of the pool. The instep of the medial arches had not been in contact with the pool surface and was not affected.

Correlation of the patient’s history and the clinical examination established the diagnosis of pool toes--a pool-associated friction-induced pedal dermatosis caused by contact of the girl’s bare feet with the bottom surface of the pool. The girl was able to achieve some relief of the pain by repeated soaking her feet in cold water; within a week, her symptoms and the plantar lesions had completely resolved.

## Discussion

Pool-associated pedal dermatoses develop in swimmers. Some commonly occur in sports participants, whereas others are seldom observed. They include asteatotic dermatitis, contact dermatitis, friction-induced injury, infections, keratoderma, pruritus, thermal injury, ultraviolet radiation exposure-related conditions, and urticaria (Table 1) [1-20]. 

**Table 1:** Pool-associated pedal dermatoses

Etiology	Condition	References
Asteatotic dermatitis	Xerosis	[1]
Contact dermatitis: allergic	Pool water dermatitis, swim fin dermatitis	[2-3]
Contact dermatitis: irritant	Pool dermatitis	[4]
Friction-induced injury	Pool toes, subcorneal hematoma	[5,6]
Infection: bacterial	Erythrasma, gram-negative toe web infection, pitted keratolysis, pseudomonas hot-foot syndrome, toe web infection	[7-10]
Infection: fungal	Dermatophyte infection, erosio interdigitalis blastomycetica, toe web infection	[10-13]
Infection: mycobacterial	Mycobacterium abscessus hand-and-foot disease	[14]
Infection: viral	Plantar verruca	[11,15]
Keratoderma	Aquagenic keratoderma	[16]
Pruritus	Aquagenic	[17]
Thermal injury	Pool feet	[18]
Ultraviolet radiation exposure	Actinic keratosis, basal cell carcinoma, drug-induced photosensitivity, melanoma, squamous cell carcinoma, sunburn	[15]
Urticaria	Aquagenic, cold-induced, contact	[17,19,20]

Swimmer’s xerosis is a form of asteatotic dermatitis that presents with dryness, itching, and scaling of the skin, including the feet. Symptoms are more pronounced in the cooler winter months, and the dryness is exacerbated when the swimming is followed by a prolonged, hot shower. The development of xerosis occurs when water evaporates from the outer layers of the skin; in addition, water dilutes the skin-produced sebum [1].

To treat and prevent swimmer’s xerosis, the swimmer should apply an emollient to the skin after either before and/or after swimming. They should also avoid long showers after completion of the swim. In addition, the application of a topical corticosteroid may be necessary if the xerosis persists [1]. 

Contact dermatitis to the feet of pool swimmers can either be allergic or irritant in etiology. Allergic contact dermatitis includes pool water dermatitis and swim fin dermatitis [2,3]. Irritant contact dermatitis includes pool dermatitis [4].

Pool water dermatitis is an allergic contact dermatitis to chemicals that are added to the pool water; it can affect the skin exposed to the pool water, including the feet. Daily treatment, by adding sanitizing chemicals, is typically performed in public pools to destroy and prevent microbial growth. Some swimmers develop eczematous dermatitis to the chemicals used in the water treatments [1,2]. 

Pool water dermatitis has occurred after exposure to compounds containing chlorine and bromine: 1-bromo-3-chloro-5,5-dimethylhydantoin. It has also been observed to agents used to lower the pH of the swimming pool: sodium metabisulfite. In addition, pool water allergic contact dermatitis has been observed to potassium peroxymonosulfate--an oxidizing agent used for shock treatment of swimming pools. Currently, there are not any prophylactic agents to reduce the risk of pool water dermatitis; therefore, affected swimmers should avoid swimming in halogenated water [1,2].

Swim fin dermatitis is an allergic contact dermatitis to the rubber fins that some swimmers put on their feet when they swim. Specifically, the swimmers may develop a reaction to either the rubber-processing accelerator (such as dibutylthiourea or diethylthiourea) or a rubber antioxidant (n-isopropyl-n’-phenyl-p-phenylenediamine). Affected individuals typically develop dermatitis several hours after wearing the fins [1,3].

Rubber-sensitized swimmers may still want to wear swim fins. Therefore, to prevent swim fin dermatitis, they should wear fins that do not contain the sensitizers. Alternatively, wearing clothing on their feet--that does not allow direct contact of the fin with their foot--may be sufficient to prevent dermatitis [1,3].

Pool dermatitis is an irritant contact dermatitis; this is in contrast to pool water dermatitis, which is an allergic contact dermatitis to some of the same chemicals that have been added to the pool water. Most of the reports of pool dermatitis have been described by Dr. Rycroft and/or Dr. Pennys. Pool dermatitis has been observed following exposure to pools that have been treated with either chlorine or 1-bromo-3-chloro-5,5-dimethylhydantoin; however, the incidence was increased in swimmers exposed to the latter chemical [4].

Frictional pool-associated pedal dermatoses include pool toes and subcorneal hematoma. These conditions result from repetitive rubbing of the affected area on the rough surface of the pool bottom or the side of the pool. Abrasion with accompanying tender erythema or a subcorneal split in the epidermis with infiltration of erythrocytes results in either pool toes or subcorneal hematoma, respectively [5,6]. 

Pool toes is a friction-related dermatitis caused by the repetitive contact of the toes--alone or with the remainder of the plantar foot--with the rough cement on the bottom of the pool. It is observed more commonly in children since many public pools have a rough cement bottom in the shallow areas in order to prevent the young swimmers who are walking in that area from slipping and falling. It also occurs more frequently early in the swim season prior to the swimmer’s feet having had an opportunity to acclimate to the pool bottom’s rough cement by thickening and/or forming calluses [5].

Most of the patients reported with pool toes have been children. However, pool toes could potentially occur in aquatic athletes who participate in pool sports and thereby affect their performance. Yet, competitive swimmers, divers, and water polo players typically do not contact the bottom of the pool with their feet, and the pools in which they practice and compete usually do not have rough bottoms [5].

Pool toes present as tender erythema of the distal plantar toes and feet. Swimmers with more severe friction-associated activity between their feet and the pool bottom may develop blisters; however, the elevated instep is spared, and the thicker heel may or may not be affected. The differential diagnosis of pool toes includes contact dermatitis, erythromelalgia, pool feet, Raynaud’s disease, and symmetric lividity of the soles [5].

Pool toes can appear after the initial exposure to the rough pool bottom or following repetitive swimming episodes during which there has been contact with the bottom of the pool. Similar lesions appearing on the ventral fingers and hands of children have been referred to as pool palms. Some of the reported patients with pool palms also concurrently had pool toes [1,5].

The symptoms and lesions of pool toes spontaneously resolve once contact between the patient’s feet and the rough cement on the pool’s bottom is eliminated. Treatment for pool toes includes soaking the feet in cold water. Also, topical therapy with a lotion containing camphor and menthol--either cooled or at room temperature--may provide some pain relief; in addition, oral acetaminophen or a nonsteroidal anti-inflammatory medication such as ibuprofen may be helpful to alleviate the tenderness of the affected areas [5].

Pool toes are typically self-limited. However, repeat exposure of the plantar toes and soles of the feet to the pool bottom can result in recurrence of the condition. Therefore, wearing rubber-soled pool shoes as protective footwear can prevent the development or recurrence of pool toes [5].

Patients with pool toes may be at increased risk to develop a bacterial infection or viral infection, such as plantar verruca, or both. They can occur since the normal intact skin has been disrupted by the friction-induced injury to the plantar toes and soles of the feet. If the abrasions are severe or blisters are present, topical antibiotic ointment may be useful to prevent bacterial infection [5].

The child in this report had erythema and superficial blisters on the cutaneous areas of her plantar feet that came in contact with the bottom surface of the pool. The affected areas included the skin overlying her metatarsals and distal phalanges and, to a lesser degree, the heel of the ventral feet; her friction-induced injury resolved spontaneously. In addition to the reported patient, other swimmers have developed pool toes after walking or doing aqua exercises in a swimming pool with the same rough bottom surface [5].

Friction between the pool edge and the feet can result in the development of a subcorneal hematoma in which blister formation and blood sequestration occur in the epidermis just below the stratum corneum. The French term “talon noir” (which translates to black heel) is often used to describe this friction-induced phenomenon. However, a diverse list of names in addition to talon noir (or black heel), based on the etiology or location or both, have been used to describe this occurrence; some of these include basketball heel, black palm, black palmar macules, calcaneal petechiae, disseminated punctate intraepidermal hemorrhage, hyperkeratosis hemorrhagica, pigmented palmar petechiae, plantar pseudochromohidrosis, play station thumb, post-traumatic punctate hemorrhage of the skin, purpura traumatica pedis, subcorneal hematoma, tennis heel, and tennis toe [6,15].

Asymptomatic, symmetric, lenticular, light brown macules developed on the pressure points located on the central plantar surfaces of a nine-year-old boy’s toes. The brown lesions appeared in the summer; they were preceded by the boy repetitively jumping into a swimming pool. The investigators determined that the brown toes were created by the friction between the boy’s feet against the rough, non-slip edge of the pool [6].

Infections of the feet of pool swimmers can be bacterial, fungal, mycobacterial, or viral. Bacterial infections include erythrasma, pitted keratolysis, pseudomonas hot-foot syndrome, and toe web infection [7-10]. Fungal infections include erosio interdigital blastomycetica, dermatophyte infections of the skin and/or toes nails, and toe web infection [10-13]. Mycobacterial infection, caused by *Mycobacterium abscessus*, can result in a hand and foot disease [14]. A plantar verruca is a human papillomavirus infection that may be more common on the feet of swimmers [12,15].

Erythrasma is a *Corynebacterium minutissimum* infection that occurs in moist areas of the body; the causative pathogen can be found in the stratum corneum of infected individuals. In addition to the axillary and inguinal folds, the toe webs are a common site of infection. The lesions appear as asymptomatic, red to brown, scaly plaques [7,15].

The suspected diagnosis of erythrasma can be confirmed using a Wood lamp. This is a black light source that emits light between 320 and 400 nanometers. The *Corynebacteria* make porphyrins (predominantly coproporphyrin III and to a lesser extent uroporphrin I) that show a bright coral-pink fluorescence during the Wood lamp examination [7].

Erythrasma can be treated systemically or topically. Effective systemic antibiotics include clarithromycin, erythromycin, and tetracycline. Topical agents that have been successfully used include clindamycin, fusidic acid, and Whitfield’s ointment (which consists of benzoic acid and salicylic acid); recently, twice daily application of 2% mupirocin ointment was also observed to be effective in resolving the infection [7].

Pitted keratolysis is a bacterial infection that occurs in the stratum corneum of the plantar feet. The development of the infection is typically preceded by hyperhidrosis and/or exposure to water, such as swimming in a pool. The infection is caused by *Corynebacterium *species, or *Dermatophilus congolensis* or *Kytococcus sedentarius* (which was formerly referred to as *Micrococcus sedentarius*) [1,8,15].

Pitted keratolysis presents as small pits or ringed erosions of the plantar stratum corneum. They are often malodorous and are located predominantly on weight-bearing areas of the feet. The infection can be treated with either systemic antibiotics (such as cephalexin, clindamycin, or erythromycin) or topical antibiotics (such as erythromycin, fusidic acid, neomycin, or clindamycin--either alone or combined with benzoyl peroxide); also, similar to erythrasma, 2% mupirocin ointment has been observed to be effective in resolving the infection [8].

Pseudomonas hot-foot syndrome (which has also been referred to as Pseudomonas hot hand-foot syndrome) is a distinct syndrome that usually occurs in children after having been exposed to water in a swimming pool (or hydrotherapy pool or hot tub or sauna or wading pool) that has a high concentration of *Pseudomonas aeruginosa*. The bacteria cause symptoms localized to the mechanically stressed areas of the plantar foot. The affected individual presents with exquisitely painful erythematous nodules on the plantar feet; walking may also be painful [9]. 

Symptoms and lesions of Pseudomonas hot-foot syndrome occur within hours to days after exposure to bacteria-containing water. The clinical differential diagnosis includes palmoplantar eccrine hidradenitis. Depending on the severity of the condition, topical antibiotics or systemic antibiotics or both have been used to treat the affected individual [1,9].

Similar to Pseudomonas hot-foot syndrome, Pseudomonas folliculitis results from aquatic exposure to *Pseudomonas aeruginosa*-contaminated water. In contrast to Pseudomonas hot-foot syndrome, Pseudomonas folliculitis lesions present as follicular-based pustules; hence the plantar feet are never affected, and rarely lesions may occur on the dorsal feet in individuals who have hair-bearing skin in that area. However, in one study, some of the affected children had concurrent lesions of Pseudomonas hot-foot syndrome and Pseudomonas folliculitis [9].

Toe web infection may be bacterial, fungal, or mixed. *Pseudomonas aeruginosa* may be the culprit; however, other gram-negative bacteria such as *Acinetobacter*, *Alcaligenes*, *Erwinia*, and *Proteus *species may be pathogens. Gram-positive bacteria--such as *Staphylococcus aureus*, group A beta-hemolytic streptococcus, and *Corynebacterium minutissimum* (which is associated with erythrasma)--can also cause toe web infection. In addition, coagulase-negative staphylococcal organisms-considered to be contamination from skin flora-may also be cultured [10].

In a study of 84 patients (56 men and 28 women) ranging from 16 to 89 years old, 78% of the individuals had bilateral toe web infection. The fourth webspace was the most common site of infection in 33% of the study participants. A predisposing factor for the development of toe web infection was swimming in a swimming pool [10].

Appropriate systemic antibiotic therapy should be initiated for the treatment of bacterial toe web infection; a bacterial culture may be helpful not only in identifying the pathogen but also for defining the organism’s susceptibility to antimicrobial agents. For patients without antibiotic allergies, empiric treatment of *Pseudomonas aeruginosa*, methicillin-susceptible *Staphylococcus aureus*, methicillin-resistant *Staphylococcus aureus*, and group A beta-hemolytic streptococcus might include a fluoroquinolone (such as ciprofloxacin), a first-generation cephalosporin (such as cephalexin), either trimethoprim-sulfamethoxazole, doxycycline, or clindamycin, and penicillin or amoxicillin, respectively.

Patients with mixed (bacterial and fungal) infection should be treated for both organisms. Therapy should not only consist of a systemic antibiotic for the bacterial infection but also an antimycotic agent administered either topically, or orally, or both. Wearing sandals or footwear while on the pool deck and public shower floors may be helpful to prevent acquiring pool-associated toe web infection [1,10,15].

Dermatophyte infections of the skin (tinea pedis) and the toenails (onychomycosis) have an increased risk of occurrence in swimmers and swimming pool employees. An examination for tinea pedis was performed on a random sample of 773 of 8291 bathers (9.3%) at a public swimming pool over a period of one week. The incidence of tinea pedis was 8.5%, and the investigators from Europe concluded that the fungal infection spread within the swimming pool [11].

Another group of researchers from Israel evaluated the prevalence of tinea pedis and onychomycosis in 169 swimming pool employees at 21 swimming pools in the Netanya area. They found that swimming pool employees, compared to controls, were 20 times more likely to have both onychomycosis and tinea pedis, 15 times more likely to only have tinea pedis, and three times more likely to have only onychomycosis. They concluded that swimming pools were a bonified source of fungal organisms [12].

Erosio interdigitalis blastomycetica is a Candida infection that affects the web spaces between the fingers or the toes that typically only occur in adults. Predisposing factors are diabetes mellitus and exposure to water. The condition occurs in areas of friction between the toes [13]. 

The morphologic presentation of erosio interdigitalis blastomycetica initially appears as a round to oval area of white and macerated tissue. In contrast to the fingers, the macerated white superficial epidermal tissue of toe erosio interdigitalis blastomycetica may not separate from the layers of the epidermis beneath it since the thicker feet skin retains its integrity. If detached, the underlying tissue is eroded and red; the clinical differential diagnosis includes erythrasma, irritant contact dermatitis, psoriasis, and toe web infection of bacterial or fungal or mixed etiology [13]. 

Toe web infection can be caused by a dermatophyte superficial fungal organism such as *Epidermophyton floccosum*, *Trichophyton mentagrophytes*, or *Trichophyton rubrum*. In this situation, the toe web infection has also been referred to as foot intertrigo or toe web intertrigo. Some affected individuals have a mixed infection of bacteria and fungi [1,10,15].

*Mycobacterium abscessus* is an environmental atypical mycobacteria. It is found in the soil and water. It is a rapidly growing organism in the laboratory [14].

*Mycobacterium abscessus* hand-and-foot disease is an uncommonly reported mycobacterial infection of wading pool or swimming pool participants. The mycobacteria were cultured not only from some of the patients but also from the pool equipment; in one outbreak, both *Mycobacterium abscessus* and *Mycobacterium abscessus/chelonae* were cultured. The outbreaks predominantly involve children and have been described in Canada, Italy, and the United States [14].

The clinical manifestations of the infection present as multiple, red to purple, lesions. The morphology of the lesion includes macules, firm papules and nodules, and pustules. The lesions are either painless or tender, painful nodules, papules, macules, and pustules on the palms and soles [14].

There was a significant delay from the time of exposure to the mycobacteria to the onset of clinical symptoms-this ranged from as early as seven to 14 days to as late as two or three months. Some of the children’s infections resolved spontaneously; however, many were treated with clarithromycin or an alternative systemic antibiotic. In addition, remediation to the swimming facility--including the pool surfaces and equipment--was performed [14].

Human papillomavirus can result in the development of plantar verruca. Plantar warts more frequently occur in swimmers. The virus may be transmitted from the swimming pools or the decks surrounding the pool [1,11,15].

Plantar verruca may be painful--either spontaneously or following pressure to the lesion; alternatively, they may be asymptomatic. They present as firm, endophytic or exophytic, plaques on ventral or lateral toes, sole, or heel of the foot. Black dots, representing thrombosed capillaries, may be visualized on the surface of the wart [1,15].

In a study of 773 swimmers, verruca was observed in 37 individuals (4.8%): two of 265 men over the age of 16 years (0.76%) and 35 of 508 children under the age of 16 years (6.9%, with a p ≤ 0.001). When the 35 children were analyzed, the incidence of foot warts ranged from 10.5% (23 of 220) in girls to 4.2% (12 of 288) in boys. The investigators concluded that swimming pools were involved in the spread of plantar verruca [11].

Aquagenic keratoderma has been observed not only as an inherited condition (with its onset in infancy and associated with either an autosomal recessive or autosomal dominant pattern of inheritance) but also as an acquired sporadic disorder often having its onset during the patient’s teens or twenties. It was originally described as an autosomal dominant disorder named hereditary papulotranslucent acrokeratoderma. Subsequently, it was reported as an acquired condition and referred to as transient reactive papulotranslucent acrokeratoderma. Several other nomenclatures have also been used to designate the condition: acquired aquagenic papulotranslucent acrokeratoderma, aquagenic palmoplantar keratoderma, aquagenic syringeal acrokeratoderma, aquagenic wrinkling of the palms, transient aquagenic hyperwrinkling, and watersports hands [16,18].

Aquagenic keratoderma has also been associated with various medications such as aminoglycosides, salazopyrin, and cyclooxygenase-2 (COX-2) inhibitors: aspirin, celecoxib, indomethacin, and rofecoxib. However, and perhaps most importantly, aquagenic keratoderma is a clinical marker for cystic fibrosis; it occurs in 40 to 80 percent of cystic fibrosis patients and in 25 percent of carriers of the disease. Other diseases associated with aquagenic keratoderma include atopic dermatitis, focal hyperhidrosis, marasmus, nephrotic syndrome, and the Raynaud phenomenon [16,18].

Hence, not only the hands but also the feet of swimmers affected by this condition will exhibit symptoms. The palms and soles will develop edema, skin wrinkling, and white to translucent papules and plaques--with or without desquamation--after exposure to water. Although the affected areas of some patients are asymptomatic, other individuals experience pruritus or burning and pain [16,18].

The pathogenesis of aquagenic keratoderma remains to be established. The common pathway involves increased skin permeability to water. This may result from either a decreased skin barrier function from dermatitis or an increase in sweat salt concentration from cystic fibrosis, drugs, or hyperhidrosis. In addition, aquaporins (which are also referred to as water channels since they function to facilitate water transport between cells by forming pores in the cell membranes) and palmar or plantar skin vasculature malfunction resulting from nerve dysfunction have been hypothesized to potentially have a role in the etiology of aquagenic keratoderma [16,17].

The clinical manifestations of aquagenic keratoderma gradually resolve within two to 20 minutes. Avoidance of water immersion is recommended for individuals with this condition. For those patients with associated hyperhidrosis, topical therapy with solutions containing aluminum salts, iontophoresis, and intracutaneous injections of botulinum toxin A may be efficacious [16].

Aquagenic pruritus presents with severe pruritus and/or burning, prickling, and stinging sensations but without any visible skin lesions; it occurs within 30 minutes after contact with any water--regardless of salinity or temperature--and can last for up to two hours. Aquagenic pruritus can be idiopathic, medication-induced (following use of bupropion, chloroquine, clomipramine, hydroxychloroquine, or testosterone), or associated with systemic diseases (such as essential thrombocytopenia, Hodgkin’s disease, hypereosinophilic syndrome, iron deficiency, juvenile xanthogranuloma, myelodysplastic syndrome, polycythemia vera, and sarcoidosis). In addition, a variant of aquagenic pruritus can occur in elderly individuals--usually older than 60 years of age--who have dry skin; showering elicits the pruritus, which is often more severe in women during the winter [1,17]. 

The pathogenesis of aquagenic pruritus has not been definitively established. Mast cell dysfunction, increased perieccrine gland fibrinolytic activity, nerve fiber acetylcholinesterase activity, and histamine and acetylcholine imbalance resulting from percutaneously absorbed factors secondary to hydration of the stratum corneum are all proposed etiologies. The potential therapeutic interventions for aquagenic pruritus are also similar to those used for patients with aquagenic urticaria [1,17].

Pool feet is a thermal injury to the soles of the feet and the plantar aspects of the toes. It is caused by contact of the patient’s feet with the hot surface surrounding the pool for an adequate duration of time to result in a burn. Similar to the pathogenesis of beach feet in which the source of heat is the sand, the ultraviolet radiation from the sun causes the tile or cement at the pool’s edge to become sufficiently hot to cause the subsequent thermal injury after contact with the individual’s feet [18].

When the ambient temperature is 75°F, beach sand can be over 100 degrees. If the skin is directly exposed to temperatures exceeding 111°F, the cellular proteins can become denatured and coagulated resulting in keratinocyte injury and manifesting as a burn. Therefore, when the outside temperature is 90°F, sand or tile or concrete temperature can be over 120°F [18].

Pool feet is a descriptive term that includes the associated location (swimming pool) and site (exposed plantar feet) of the thermal injury. A similar injury would result from stepping with bare feet on asphalt pavement, metal, plastic, or rubber that has become hot from sunlight or an alternative heat source. Indeed, similar to the sand at the beach, the tiles around a pool can become hot enough to result in first-degree or second-degree burns [18].

First-degree burns are superficial and are characterized by pain and redness; they are limited to the epidermis. Second-degree burns involve partial thickness of the skin and present as painful blisters or erosions; they extend into the dermis. Third-degree burns affect the full thickness of the skin and are asymptomatic; they extend into the fat beneath the skin [18].

The dorsal feet of not only swimmers but also individuals socializing at pools who are either bare-footed or have footwear that partially covers their skin are susceptible to acute phototoxic skin injury (sunburn) from the ultraviolet radiation emitted from the sun. In addition, pool participants who are on certain medications can be susceptible to drug-induced photosensitivity--either phototoxic or photoallergic drug reactions. Chronic sun exposure can result in actinic keratoses, non-melanoma skin cancer (such as basal cell carcinoma and squamous cell carcinoma), and melanoma [1,15,18].

Urticaria presents as pruritic erythematous wheals. The lesions are variable in size and location. Typically, urticarial lesions can recur daily but resolve spontaneously within 24 hours; however, in patients with physical urticarias, exposure to the inducing stimulus will elicit recurrence of the lesions. Swimming in fresh or chlorinated water has been observed to cause not only aquagenic urticaria but also, albeit less frequently, cold-induced urticaria and contact urticaria [1,17,19,20].

Aquagenic urticaria is a rare physical urticaria that presents as one millimeter to three millimeter folliculocentric wheals surrounded by one centimeter to three centimeters of erythema within 20 to 30 minutes after the skin has contacted any water. The skin lesions may be associated with pruritus or a sensation of either burning or prickling or pruritus; rarely, there can be systemic symptoms. The upper arms and trunk are the most common lesion location; although the palms and soles are usually spared, the lesions may appear on the dorsal aspect of the hands and the feet [1,17].

Most cases of aquagenic urticaria are sporadic; however, a few familial cases--either in identical twin brothers or in patients with a systemic disease (such as Bernard-Soulier syndrome or lactose intolerance)--have been observed. Hence, although there may be a genetic component to the etiology of aquagenic urticaria, the pathogenesis of this condition still remains to be determined. Hypotheses include a reaction between the water and sebum forming a substance that causes mast cell degranulation, a water-soluble epidermal antigen that enters the dermis and causes histamine release, and urticaria being triggered by an increased passive diffusion of water resulting from a sudden change in perifollicular osmotic pressure [17].

The first line of treatment for aquagenic urticaria is antihistamines. Other therapeutic interventions include anabolic androgenic steroids (such as stanozolol), phototherapy (using ultraviolet B radiation or psoralen and ultraviolet A radiation), selective serotonin reuptake inhibitors (such as fluoxetine), and topical barrier creams. Omalizumab (300 milligrams every 28 days) resulted in improvement after two injections in a patient with both aquagenic urticaria and aquagenic pruritus [1,17].

Cold-induced urticaria can have a genetic component (familial autoinflammatory syndrome, which is a cryopyrin-associated periodic syndrome) or acquired. Acquired cold-induced urticaria can be idiopathic (or essential) and may be associated with atopy. However, secondary causes of acquired cold-induced urticaria include cryoglobulinemia and connective tissue diseases [1,19].

Acquired cold-induced urticaria presents with pruritic erythematous hives or angioedema or both following exposure to cold. Importantly, in a retrospective study of 415 children with acquired cold-induced urticaria, nearly a fifth of the children (18.6%) experienced severe reactions (such as generalized urticaria and/or angioedema with systemic manifestations including airway, respiratory, abdominal, cardiovascular, and/or neurologic symptoms). Swimming triggered 77.6% of these reactions [19]. 

A cold stimulation test is used to attempt to establish the diagnosis of cold-induced urticaria. Ice-cube challenge testing is performed by placing a plastic bag containing an ice cube on the volar surface of the arm for five minutes and allowing for five to 10 minutes of rewarming; the presence of a wheal over the application site is considered a positive test. If the test is negative, it can be repeated using 10 minutes of cold ice cube contact [19]. In the pediatric patient study, an increased risk of anaphylaxis was associated with a positive cold stimulation test; however, an 11.7% rate of anaphylaxis was observed in children with a negative test [19].

Treatment of acquired cold-induced urticaria in the pediatric patient study included maintenance therapy with H1-blocker antihistamines. Other medications that some of the children received were montelukast, an H2-blocker antihistamine, oral corticosteroids, doxepin, or omalizumab. Also, the patients and their families were counseled about the risks and triggers for anaphylaxis and received a prescription for an epinephrine autoinjector [19].

Contact urticaria in swimmers has rarely been reported. An otherwise healthy 35-year-old sickbay attendant of the Royal Netherland Navy would develop intermittent urticaria after one to five hours of swimming in a pool containing chlorinated water; sodium hypochlorite was used to disinfect the pool water. He did not develop urticaria after swimming in freshwater or seawater. Also, he experienced no problems drinking water chlorinated with calcium hypochlorite while onboard ship [20].

However, urticaria occurred 30 minutes after non-intentional contact with a cleaning powder containing sodium hypochlorite. Closed patch testing with sodium hypochlorite and calcium hypochlorite was performed; although evaluation after 20 and 60 minutes was negative, extensive urticaria was observed when the patch testing sites were examined after three hours. The investigators concluded that in patients suspected of aquagenic urticaria, the possibility of contact urticaria from chlorinated swimming pool water should be considered [20].

## Conclusions

There are several pool-associated dermatoses of the feet that may occur in participants who swim. The clinical features of a girl who developed a friction-induced injury to her plantar feet, pool feet, are described. In addition to pool toes, asteototic dermatitis, contact dermatitis, other friction-induced injury, infections, keratoderma, pruritus, thermal injury, ultraviolet radiation exposure-related conditions, and urticaria are other pedal conditions that can occur in freshwater pool swimmers.
